# Sinus rhythm restoration reverses tricuspid regurgitation in patients with atrial fibrillation: a systematic review and meta-analysis

**DOI:** 10.1186/s13019-024-02891-9

**Published:** 2024-07-02

**Authors:** Yufeng Zhan, Ning Li

**Affiliations:** 1 Department of Anesthesia, Affiliated Hangzhou First People’s Hospital, School of Medicine, Westlake University, Hangzhou, 310000 China; 2 Department of Cardiothoracic Surgery, Naval Medical Center of PLA, Naval Medical University, Shanghai, 200052 China

**Keywords:** Tricuspid regurgitation, Atrial fibrillation, Sinus rhythm restoration, Ablation, Cardiac remodeling

## Abstract

**Background:**

Tricuspid regurgitation (TR) is a common valvular heart disease worldwide, and current guidelines for TR treatment are relatively conservative, as well as with detrimental outcomes. Restoration of sinus rhythm was reported to improve the TR severity in those TR patients with atrial fibrillation (AF). However, relevant research was limited. The aim of this meta-analysis was to evaluate the clinical outcomes of restoration of sinus rhythm in TR patients with AF.

**Methods:**

In this study, PubMed, Web of Science, and Scopus databases were searched for study enrollment until July 2023. This study was designed under the guidance of Preferred Reporting Items for Systematic Reviews and Meta-Analyses. These studies containing the patient’s baseline characteristics, surgical procedure, and at least one of the clinical outcomes were included. The primary endpoint was TR grade during follow-up after restoration of sinus rhythm.

**Results:**

Out of 1074 records, 6 were enrolled. Restoration of sinus rhythm is associated with a reduction of TR severity (TR grade, odds ratio 0.11, 95% confidence interval (CI): 0.01 to 1.28, *P* = 0.08, I^2^ = 83%; TR area, mean difference (MD) -2.19 cm^2^, 95% CI: -4.17 to -0.21 cm^2^, *P* = 0.03, I^2^ = 96%). Additionally, remolding of right heart with a significant reduction of tricuspid valve annulus diameter (MD -0.36 cm, 95%CI: -0.47 to -0.26 cm, *P* < 0.00001, I^2^ = 29%) and right atrium volume index (MD -11.10 mL/m^2^, 95%CI: -16.81 to -5.39 mL/m^2^, *P* = 0.0001, I^2^ = 79%) was observed during follow-up.

**Conclusions:**

In conclusion, rhythm-control therapy could reduce TR severity in AF patients with TR and is associated with right heart remodeling.

**Supplementary Information:**

The online version contains supplementary material available at 10.1186/s13019-024-02891-9.

## Introduction

Tricuspid regurgitation (TR) is a common valvular heart disease worldwide, which has a high incidence of right heart failure and hospitalization for heart failure, as well as a high rate of complication and mortality [1]. A population-based cohort of patients with AF showed that nearly one-third developed moderate or greater TR over time [2]. The current guidelines for TR treatment are relatively conservative, mainly based on comprehensive treatment with diuretics. Isolated tricuspid valve surgery has higher mortality and morbidity than left-sided valve surgery, with perioperative mortality as high as 3 ~ 11%. Late referral, progression of heart failure symptoms, and right ventricular dysfunction suggest a poor prognosis [3–6].

TR is divided into primary and secondary according to the etiology, and secondary TR occupies the predominate position, which is accompanied with tricuspid annulus dilatation and leaflet tethering. Atrial functional TR has gradually captured attention in recent years [7]. Atrial functional TR is mediated by dilatation of the right atrium, and most cases are accompanied with long-standing atrial fibrillation (AF), which is associated with dilatation of the right atrium and tricuspid annulus [8]. It is worth noting that the population proportion of TR with AF is more than 80% in some transcatheter tricuspid valve intervention cohorts [9–11].

Previous studies have proved that AF predisposes patients to TR progression [2, 7]. The severity of TR and TR jet area were significantly improved during follow-up after radiofrequency ablation to restore sinus rhythm, and the improvement of TR may be related to the reverse remodeling of the right heart [12–17]. Although the worsening TR is associated with AF, there is limited research focused on the effect of restoring sinus rhythm on the TR severity. In this meta-analysis, we aimed to systematically review and analyze the effect of sinus rhythm restoration on the severity of TR.

## Methods

### Literature search and study enrollment

**Fig. 1:**
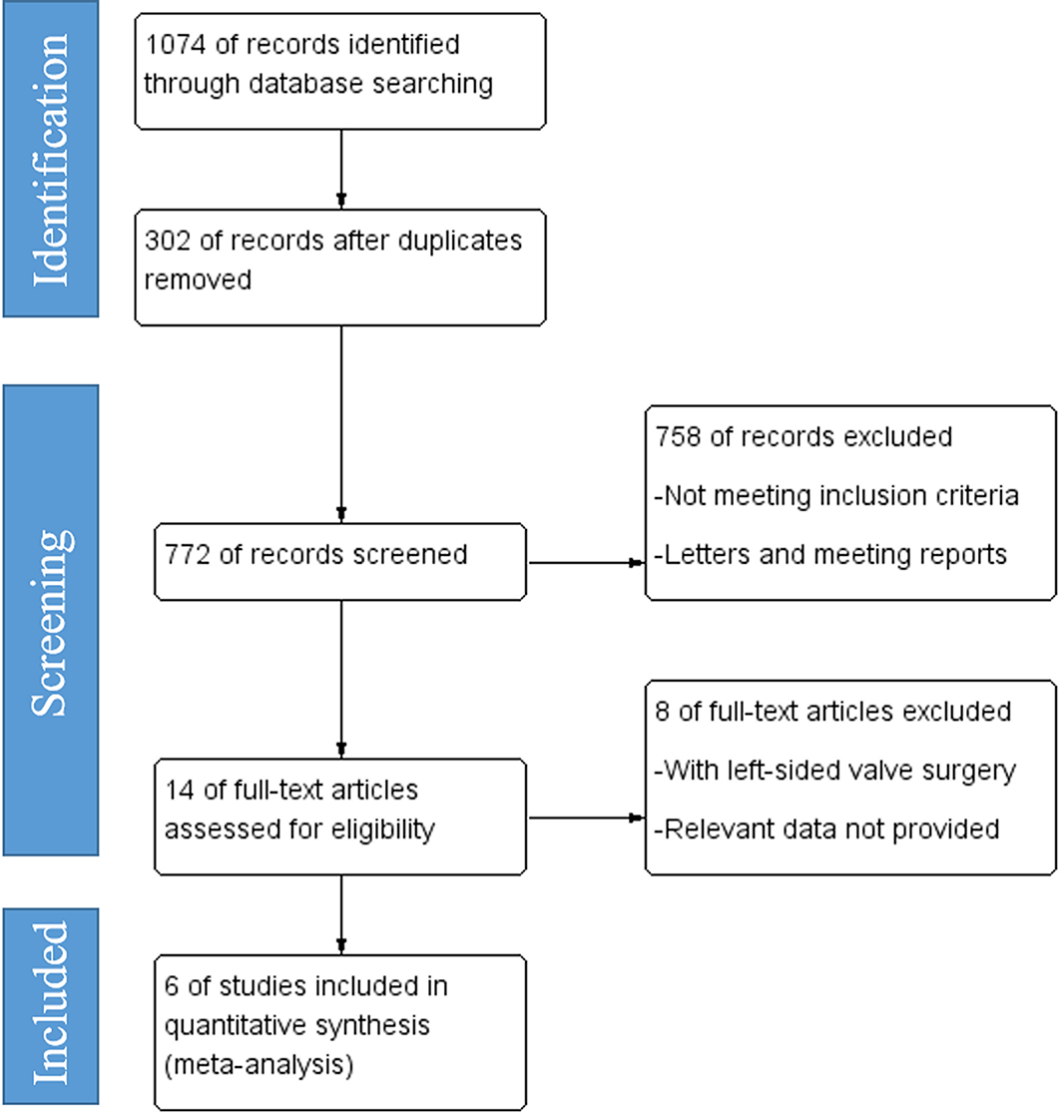
Preferred reporting items for systematic reviews and meta-analyses study flow chart diagram

This study was designed under the guidance of Preferred Reporting Items for Systematic Reviews and Meta-Analyses (PRISMA) [18]. Ethics approval and informed consent were waived in this study. Two authors systematically searched Medline/PubMed, Web of Science, Scopus, Google Scholar, and ClinicalTrials.gov up to July 2023 to evaluate the efficacy of sinus rhythm restoration in alleviating TR in AF patients. The major medical subject heading used in this study were “tricuspid valve” and “atrial fibrillation”, the intact search strategy was shown in Supplementary Materials S1. English was set as a language restriction. The original study protocol was registered on the PROSPERO platform (CRD42023441368). Two independent investigators reviewed all titles and abstracts and selected potentially eligible papers. Eligible studies were further read in detail to assess inclusion or exclusion criteria. The reference lists of included studies were also further traced to identify other studies of potential interest. Any controversy regarding the inclusion of a paper was discussed until an agreement was reached. Letters, meeting reports, and personal communications were excluded from this study (Fig. 1). The risk of bias for each included study was assessed using the Newcastle-Ottawa quality assessment scale as shown in Supplementary Materials S1.

### Inclusion criteria

Studies were enrolled when all the following criteria applied: (1) the study had to report the patient’s baseline characteristics, surgical procedure or detailed information, and at least one of the clinical outcomes we were interested in; (2) the number of enrolled patients was at least 15; (3) when the same group of patients was included in different studies, only the study containing the largest number of patients was included; (4) studies with concomitant valve surgery were excluded.

### Data extraction

Information on study design, sample size, clinical and echocardiographic characteristics of patients, duration of follow-up, and clinical outcomes of interest were extracted from the selected studies. TR severity were graded qualitatively into 3 categories: no/mild, moderate, and severe. Any discrepancies were resolved by consensus.

### Endpoints and definitions

The primary endpoint of this study was to evaluate the effect of restoration of sinus rhythm on the TR severity in TR patients with AF. The improvement of TR was defined as the patients with a residual TR grade of ≤ 1+. As secondary endpoints, we further evaluated the change in the echocardiographic changes during follow-up: tricuspid annular diameter, right atrium volume index, pulmonary artery systolic pressure, and left ventricular ejection fraction.

### Statistical analysis

The sample means and standard deviation in the original research, which was present with the formation of Median (interquartile range), were estimated by Luo et al. [19]and Wan et al. [20]. Odds ratio (OR) with 95% confidence intervals (CI) and standardized mean differences (MDs) were used as summary statistics for the comparison of dichotomous and continuous variables, respectively. A random-effects model was used to obtain the OR and MD when significant heterogeneity was detected. Otherwise, the fixed-effect model was applied. Inverse-variance and Mantel–Haenszel fixed-effect methods were used for meta-analysis. The heterogeneity is described by using I^2^ test. The values of I^2^ as 25%, 50%, and 75% represent little, moderate, and substantial heterogeneity, respectively. Statistical significance was set at *P* < 0.05. Publication bias was assessed by funnel plots. All analyses were performed by Reviewer Manager (RevMan, version 5.3).

## Results

### Identification of studies and baseline characteristics

The literature retrieval process was shown in Fig. 1, a total of 1074 records were enrolled in this study, and finally 6 studies were included in this meta-analysis. There was no significant difference in age between both restorations of the sinus rhythm group and the control group (*P* = 0.35). In addition, the male ratio in the restoration of sinus rhythm group and control group was 69.4% and 67.0%, respectively. Other relevant baseline characteristics were shown in Table 1. Successful cardioversion and/or ablation were performed in the sinus rhythm group.

**Table 1:** Baseline clinical characteristics of the included studies

Study	Markman TM et al. **(13)**	Nishiwaki S et al. **(12)**	Pype L et al. **(30)**		Itakura K et al. **(14)**		Nakatsukasa T et al. **(15)**	Soulat-Dufour L et al. **(16)**	
	Before PVI	Before PVI	AF ablation	AF	catheter ablation	AF	Before PVI	Active SR	AF
Year	2020	2022	2019		2020		2023	2022	
Country	USA	Japan	Belgium		Japan		Japan	French	
Time span	2011–2017	2004–2019	2007–2014		2011–2015		2016–2017	N/A	
Research style	Retrospective	Retrospective	Retrospective		Retrospective		Retrospective	Prospective	
Sample	*n* = 36	*n* = 64	*n* = 43	*n* = 39	*n* = 71	*n* = 15	*n* = 89	*n* = 47	*n* = 39
Age (years)	63.7 ± 11.1	73.4 ± 8.3	59 ± 11	66 ± 9	60.8 ± 9.7	58.0 ± 7.2	63.8 ± 12.1	64.1 ± 11.6	68.5 ± 12.2
Male (%)	20 (56)	31(48)	34(79)	28(72)	56 (78.9)	14 (93.3)	71 (79.0)	31 (66.0)	25 (64.1)
Type of AF, %									
Paroxysmal	17 (47)	17(27)	30(70)	28(72)	0 (0)	0 (0)	63 (70.8)	17 (36.2)	10 (26.0)
Persistent		36 (56)	0 (0)	0 (0)	0 (0)	0 (0)	13 (14.6)	0 (0)	0 (0)
Persistent	19 (53)	11 (17)	13(30)	11(28)	71(100)	15(100)	13 (14.6)	30 (63.8)	29 (74.0)
Treatment strategies	PVI	catheter ablation	catheter ablation		PVI		PVI	cardioversion and/or ablation	
Hypertension	20 (56)	34 (53)	20(47.6)	28(71.8)	35 (49.3)	10 (66.7)	49 (55.0)	18 (38.3)	25 (64.1)
Diabetes mellitus	8 (22)	3 (5)	3(7)	7(17.9)	9 (12.7)	3 (20.0)	12 (13.5)	6 (12.8)	11 (28.2)
CAD	9 (25)	N/A	8(18.6)	8(20.5)	N/A	N/A	N/A	15 (31.9)	12 (64.1)
Stroke or TIA	4 (11)	7 (11)	N/A	N/A	7 (9.9)	0 (0.0)	N/A	3 (6.4)	4 (10.3)
Follow-up length	6 months	6–18 months	60 months	73 months	12 months	12 months	3–6 months	12 months	12 months

PVI, pulmonary vein isolation; AF, atrial fibrillation; SR, sinus rhythm; CAD, Coronary artery disease; TIA, transient ischemic attack

### Clinical outcomes

**Fig. 2:**
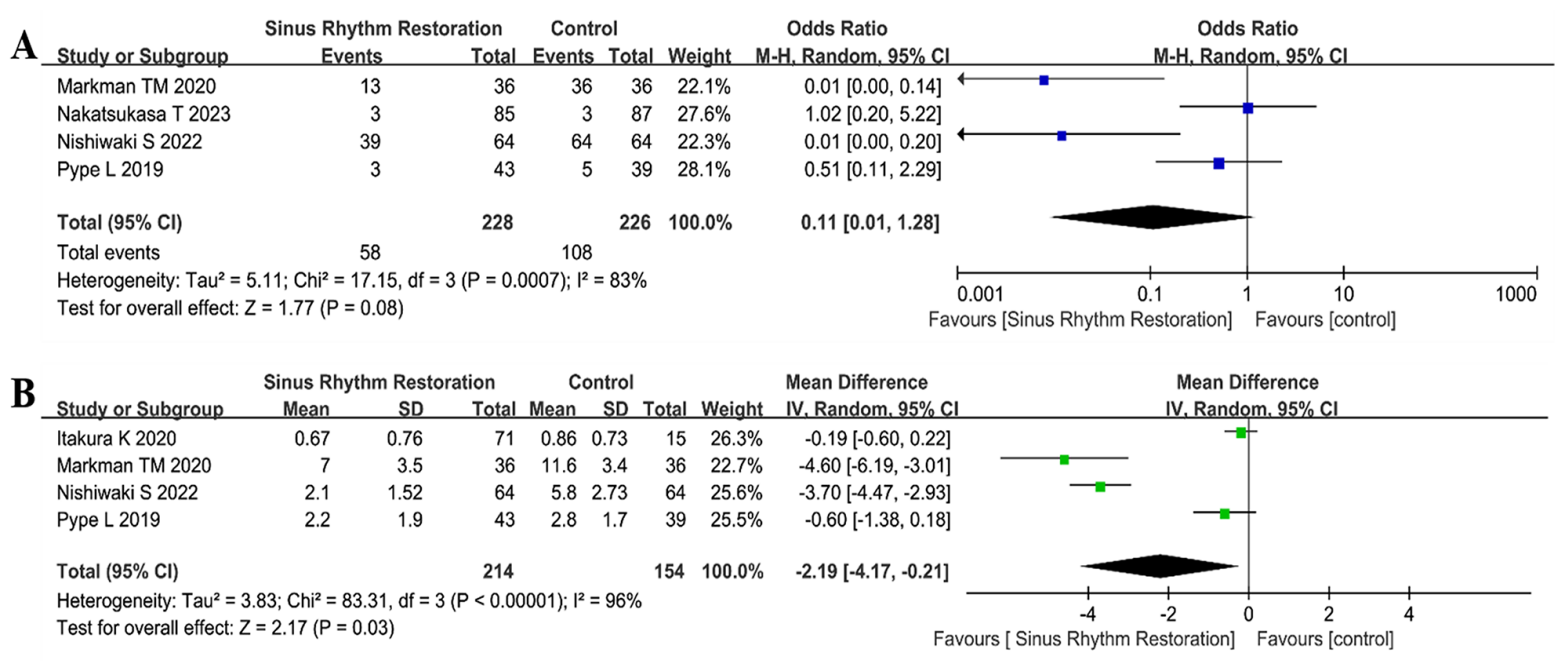
Forest plot for the severity of tricuspid regurgitation after restoration of sinus rhythm. **A**, tricuspid regurgitation grade; **B**, tricuspid regurgitation area

**Fig. 3:**
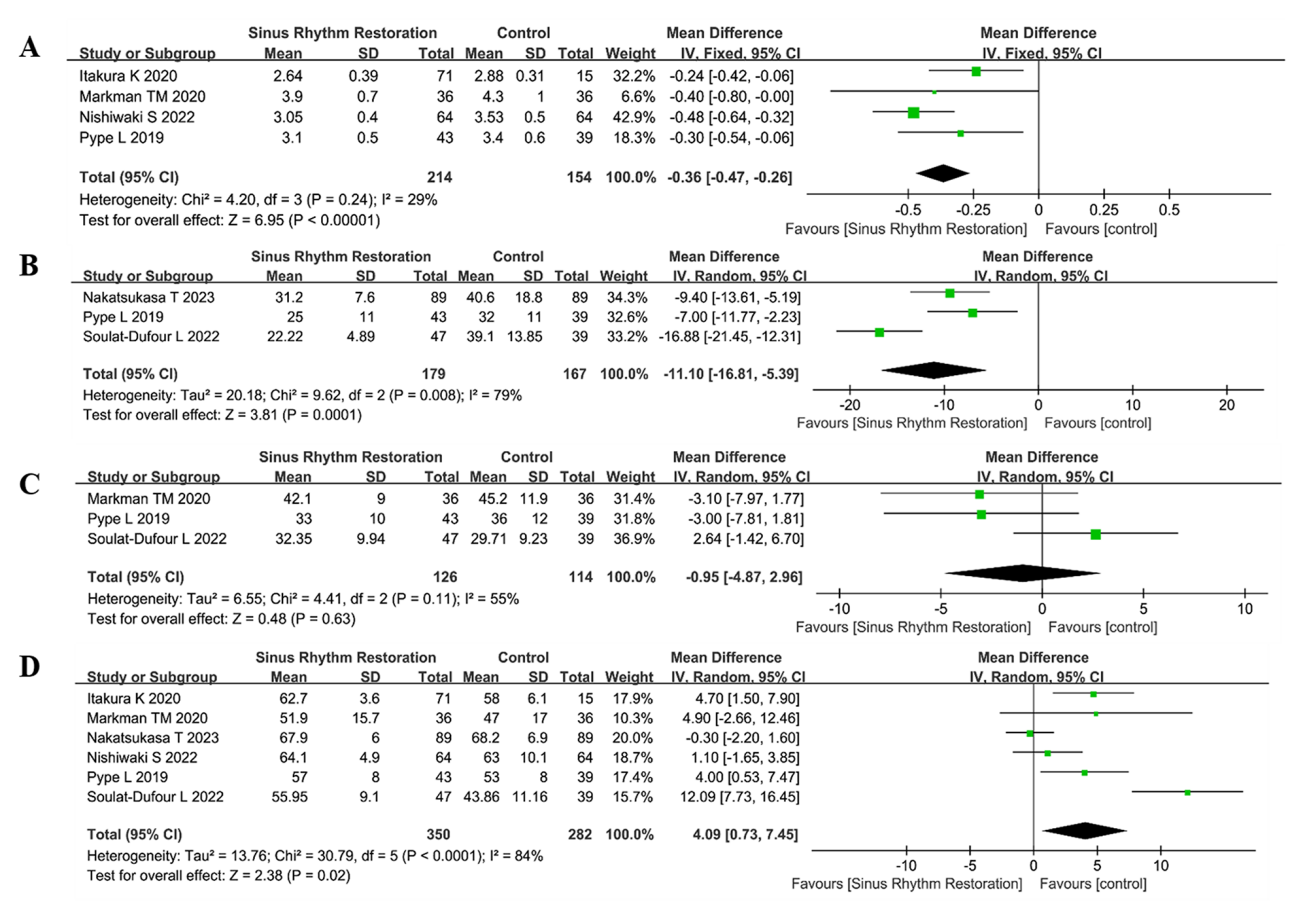
Forest plot for the assessment of echocardiographic outcomes during follow-up after restoration of sinus rhythm. **A**, tricuspid valve annulus diameter; **B**, right atrium volume index; **C**, pulmonary artery systolic pressure; **D**, left ventricular ejection fraction

The severity of TR decreased after the restoration of sinus rhythm, which was identified by measurement of TR grade qualitatively (OR 0.11, 95%CI: 0.01 to 1.28, *P* = 0.08, I^2^ = 83%, Fig. 2A). Similarly, quantitative measurement of TR with TR area significantly decreased after rhythm-control treatment (MD -2.19 cm^2^, 95%CI: -4.17 to -0.21 cm^2^, *P* = 0.03, I^2^ = 96%, Fig. 2B). A significant remolding of the right heart was observed during follow-up, which was depicted with a reduced tricuspid valve annulus diameter (MD -0.36 cm, 95%CI: -0.47 to -0.26 cm, *P* < 0.00001, I^2^ = 29%, Fig. 3A) and right atrium volume index (MD -11.10 mL/m^2^, 95%CI: -16.81 to -5.39 mL/m^2^, *P* = 0.0001, I^2^ = 79%, Fig. 3B). Of note, the pulmonary artery systolic pressure did not change significantly (MD -0.95 mmHg, 95%CI: -4.87 to 2.96 mmHg, *P* = 0.63, I^2^ = 55%, Fig. 3C), paired with a slight significant increase in left ventricular ejection fraction (MD 4.09, 95%CI: 0.73 to 7.45, *P* = 0.02, I^2^ = 84%, Fig. 3D). The detailed clinical results were shown in Table 2. The publication bias was shown in Supplementary Materials S2.

**Table 2:** Clinical results of the included studies

Study	Markman TM et al. **(13)**		Nishiwaki S et al. **(12)**		Pype L et al. **(30)**		Itakura K et al. **(14)**		Nakatsukasa T et al. **(15)**		Soulat-Dufour L et al. **(16)**	
	Before PVI	After PVI	Before PVI	After PVI	AF ablation	AF	catheter ablation	AF	Before PVI	After PVI	Active SR	AF
TR grade												
0 (%)	0(0)	0(0)	0(0)	9(14)	0(0)	0(0)	N/A	N/A	37 (42.6)	39 (45.9)	N/A	N/A
1 (%)	0(0)	23 (64)	0(0)	16(25)	42(97.6)	38(97.2)	N/A	N/A	47 (54.0)	43 (50.6)	N/A	N/A
2 (%)	33(92)	10 (28)	42(64)	31(48)	1(2.4)	1(2.8)	N/A	N/A	3 (3.4)	3 (3.5)	N/A	N/A
3 (%)	3(8)	3 (8)	22(34)	8(13)	0 (0)	0 (0)	N/A	N/A	0 (0)	0 (0)	N/A	N/A
Tricuspid valve annular diameter, cm	4.3 (1.0)	3.9 (0.7)	3.56 (3.19–3.85)	3.01 (2.78–3.36)	3.1 ± 0.5	3.4 ± 0.6	2.64 ± 0.39	2.88 ± 0.31	N/A	N/A	N/A	N/A
Tricuspid regurgitation area, cm^2^	11.6 (3.4)	7.0 (3.5)	5.8 (4.0–7.6)	2.1 (1.1–3.1)	3.1 ± 2.6	3.0 ± 2.3	0.6 (0.2–1.2)	0.9 (0.4–1.3)	N/A	N/A	N/A	N/A
right atrial volume index, ml/m^2^	N/A	N/A	N/A	N/A	25 ± 11	32 ± 11	N/A	N/A	40.6 ± 18.8	31.2 ± 7.6	22.5 ± 4.89	37.4 ± 13.8
Pulmonary artery systolic pressure, mmHg	45.2 (11.9)	42.1 (9.0)	N/A	N/A	33 ± 10	36 ± 12	N/A	N/A	N/A	N/A	32 ± 9.9	29 ± 9.2
Left ventricular ejection fraction,%	47.0 (17.0)	51.9 (15.7)	62.5 (56.6–69.9)	63.7 (61.1–67.5)	57 ± 8	53 ± 8	62.7 ± 3.6	58.0 ± 6.1	68.2 ± 6.9	67.9 ± 6.0	N/A	N/A

PVI, pulmonary vein isolation; AF, atrial fibrillation; SR, sinus rhythm

## Discussion

The natural history of the TR is associated with progressive symptoms and requires treatment with diuretics to alleviate symptoms. Isolated tricuspid valve surgery has a high risk of mortality and complications, and the evidence supporting transcatheter tricuspid valve intervention is also limited. In this study, we focused on the impact of sinus rhythm restoration on the reverse of TR severity in patients with AF. Our results suggest that restoration of sinus rhythm alleviates TR progression and reverses right heart remodeling.

TR severity and jet area, as well as mitral regurgitation jet area, are improved after catheter ablation in AF patients with significant TR after retrospectively analyzing consecutive 64 patients with functional TR in Nishiwaki S’s study [12]. Soulat-Dufour L et al. reported shrinkage of right atrium volume after catheter ablation, thereafter, multiple studies observed consistent findings with shrinkage of tricuspid valve annulus post-ablation [13, 14, 16]. In addition, the reverse remodeling of the right heart is also identified by echocardiography during follow-up, suggest that the underlying explanation of TR severity improvement. Nakatsukasa T et al. [15]pointed out that AF not only enlarges the right atrium but changes the shape and size of the tricuspid valve annulus after analyzing the tricuspid valve morphology by multi-detector row computed tomography. Rhythm-control therapy resulted in reverse remodeling of the tricuspid valve annulus and reduced right atrial volume, emphasizing the importance of early AF intervention to maintain the tricuspid valve complex structure [15]. A rhythm control strategy was advocated in the early stage since the ability to maintain sinus rhythm was inversely linked to the AF duration and enlarged right atrium volume. Sinus rhythm maintenance post-operation was proved to be the most significant determinant of TR reduction by multivariate analysis [17].

In the present study, we excluded studies in which left-sided valve surgery was combined with a Maze procedure. A significant reduction in the degree of TR has also been observed after concomitant mitral valve surgery with a Maze procedure [21–23]. Stulak JM et al. analyzed the clinical data of 33 patients who underwent mitral valve surgery with Maze surgery concomitantly and found that the degree of TR improved to 1.9 ± 0.9 (*P* = 0.078 versus preoperative) after surgery, and only 9% (3 of 33 patients) had advanced TR during follow-up. On the contrary, in patients who were without the Maze procedure concomitantly, the degree of TR deteriorated to 2.7 ± 0.9 during follow-up, and 45% (15/33) of the patients had aggravated TR. The multivariate regression analysis further confirmed that the Maze surgery was a protective factor for postoperative TR progression [23]. Similarly, Kim HK and colleagues found that 12.8% of patients who underwent left-sided valve surgery with a Maze procedure had postoperative TR deterioration, as compared with 38.8% of patients who without a Maze procedure [21].

The Maze procedure has an acceptable rate of sinus rhythm restoration and reduces long-term major adverse cardiovascular and cerebrovascular events in the treatment of severe TR and persistent AF [24]. TR patients with persistent AF who underwent tricuspid valve repair combined with Maze procedure were proved with a 10-year sinus rhythm rate of 55%, as well as with lower major adverse cardiovascular and cerebrovascular event rates at 15 years compared to those TR patients without the Maze procedure [25]. In mitral valve surgery patients with AF, performance with surgical ablation to restore sinus rhythm appears to be associated with lower risk-adjusted operative mortality than in those who do not undergo ablation [26]. Hence, a concomitant Maze procedure may act as a good choice when left-sided heart surgery is necessary.

Of note, not all patients with moderate to severe TR caused by AF benefit from rhythm control approaches. Tethering height was shown to be an independent risk factor for the late recurrence of AF and TR. TR patients with AF who are with tethering height less than 6 mm have satisfactory tricuspid valve function improvement after the restoration of sinus rhythm after catheter ablation [27]. Surgical ablation concomitant with tricuspid valve repair is advocated in TR patients with severe tethering height and less leaflets coaptation. Relatively young age and absence of late recurrence of AF are good predictors of TR improvement after successful ablation, and improvement of TR is associated with better clinical outcomes [28].

Permanent AF can induce TR through right atrium enlargement and subsequent tricuspid annulus dilation [29]. This study confirmed that continuous rhythm control was beneficial to delay the progression of TR. The effect of sinus rhythm restoration on alleviating TR is mainly driven by right heart remodeling and blocking the dilation process, which further confirms the bidirectional causal relationship between AF and TR [30]. Therefore, successful restoration and maintenance of sinus rhythm are extremely important for TR reversal, and the underlying mechanisms still need to be further studied. Both right-sided heart size and function improved after sinus rhythm restoration, and whether these improvements in size and function were a cause or a consequence of reduced TR grade requires further prospective studies in clinical or experimental models.

### Limitation

This study suffers from the intrinsic limitations of all meta-analyses, especially the selection bias on account of the non-randomized nature of the enrolled research. Secondly, the original data of enrolled studies is not available, and the data could only be analyzed secondary. Although the end effects of different studies are similar, residual unexplained heterogeneity in their therapeutic effects cannot be ruled out. The enrolled patient number in this study is limited. Thirdly, the parameters evaluating the function of the right-sided heart and TR degree are relatively simple, tricuspid annular plane systolic excursion and fractional area change, TR jet width, inferior vena cava reflux, and other parameters are not reflected. TR grade is not assessed according to the echocardiographic guideline (mild, moderate, severe, massive, and torrential). At last, data regarding long-term follow-up survival and heart failure are lacking, and patients experienced AF recurrence during follow-up are also unknown.

## Conclusion

Rhythm-control therapy could reduce TR severity in AF patients with TR and is associated with right heart remodeling.

### Electronic supplementary material

Below is the link to the electronic supplementary material.

Supplementary Material 1

Supplementary Material 2

## Data Availability

No datasets were generated or analysed during the current study.

## References

[1] Topilsky Y, Maltais S, Medina Inojosa J (2019). Burden of Tricuspid Regurgitation in patients diagnosed in the community Setting[J]. JACC Cardiovasc Imaging.

[2] PATLOLLA SH, SCHAFF HV (2022). Incidence and Burden of Tricuspid Regurgitation in patients with Atrial Fibrillation[J]. J Am Coll Cardiol.

[3] CHEN Q, BOWDISH ME (2023).

[4] SCOTTI A, STURLA M (2022). Outcomes of isolated tricuspid valve replacement: a systematic review and meta-analysis of 5,316 patients from 35 studies[J]. EuroIntervention.

[5] ELGHARABLY H, IBRAHIM A (2021). Right heart failure and patient selection for isolated tricuspid valve surgery[J]. J Thorac Cardiovasc Surg.

[6] DREYFUS J, FLAGIELLO M (2020). Isolated tricuspid valve surgery: impact of aetiology and clinical presentation on outcomes[J]. Eur Heart J.

[7] Utsunomiya H, Itabashi Y, Mihara H, Berdejo J, Kobayashi S, Siegel RJ, et al. Functional tricuspid regurgitation caused by Chronic Atrial Fibrillation: a real-time 3-Dimensional Transesophageal Echocardiography Study[J]. Circ Cardiovasc Imaging. 2017;10(1). 10.1161/CIRCIMAGING.116.004897. 10.1161/CIRCIMAGING.116.004897 28073806

[8] ORTIZ-LEON XA, POSADA-MARTINEZ EL, TREJO-PAREDES MC (2020). Understanding tricuspid valve remodelling in atrial fibrillation using three-dimensional echocardiography[J]. Eur Heart J Cardiovasc Imaging.

[9] WEI W NINGL (2022). Hemodynamics of transcatheter tricuspid valve replacement with Lux-Valve[J]. Front Cardiovasc Med.

[10] WEBB JG, CHUANG AM MEIERD (2022). Transcatheter tricuspid valve replacement with the EVOQUE System: 1-Year outcomes of a Multicenter, First-in-human Experience[J]. JACC Cardiovasc Interv.

[11] SORAJJA P, WHISENANT B (2023). Transcatheter repair for patients with tricuspid Regurgitation[J]. N Engl J Med.

[12] WATANABE NISHIWAKIS (2022). Impact of catheter ablation on functional tricuspid regurgitation in patients with atrial fibrillation[J]. J Interv Card Electrophysiol.

[13] MARKMAN TM, PLAPPERT T, DE FERIA ALSINA A (2020). Improvement in tricuspid regurgitation following catheter ablation of atrial fibrillation[J]. J Cardiovasc Electrophysiol.

[14] ITAKURA K, HIDAKA T (2020). Successful catheter ablation of persistent atrial fibrillation is associated with improvement in functional tricuspid regurgitation and right heart reverse remodeling[J]. Heart Vessels.

[15] NAKATSUKASA T, ISHIZU T (2023). Reverse remodeling of the tricuspid valve complex by sinus rhythm restoration after catheter ablation[J]. J Cardiol.

[16] SOULAT-DUFOUR L, LANG S (2022). Restoring Sinus Rhythm reverses Cardiac Remodeling and reduces valvular regurgitation in patients with Atrial Fibrillation[J]. J Am Coll Cardiol.

[17] CHA MJ, LEE SA (2023). Reduction of moderate to severe tricuspid regurgitation after catheter ablation for atrial fibrillation[J]. Heart.

[18] PAGE MJ, MCKENZIE JE (2021). The PRISMA 2020 statement: an updated guideline for reporting systematic reviews[J]. BMJ.

[19] WAN LUOD, TONG XLIUJ (2018). Optimally estimating the sample mean from the sample size, median, mid-range, and/or mid-quartile range[J]. Stat Methods Med Res.

[20] WAN X, WANG W, TONG LIUJ (2014). Estimating the sample mean and standard deviation from the sample size, median, range and/or interquartile range[J]. BMC Med Res Methodol.

[21] KIM HK, KIM YJ (2005). Impact of the maze operation combined with left-sided valve surgery on the change in tricuspid regurgitation over time[J]. Circulation.

[22] Wang J, Han J, Li Y, Ye Q, Meng F, Luo T, et al. Impact of Surgical ablation of Atrial Fibrillation on the progression of Tricuspid Regurgitation and Right-Sided Heart Remodeling after mitral-valve surgery: a propensity-score matching Analysis[J]. J Am Heart Assoc. 2016;5(12). 10.1161/JAHA.116.004213. 10.1161/JAHA.116.004213 PMC5210400 27919928

[23] Stulak JM, Schaff HV, Dearani JA, Orszulak TA, Daly RC, Sundt III TM, et al. Restoration of sinus rhythm by the Maze procedure halts progression of tricuspid regurgitation after mitral surgery[J]. Ann Thorac Surg, 2008, 86(1): 40–44; discussion 44–45. 10.1016/j.athoracsur.2008.03.004. 10.1016/j.athoracsur.2008.03.004 18573396

[24] PARK I, JEONG DS (2021). Impact of maze procedure in patients with severe tricuspid regurgitation and persistent atrial fibrillation[J]. J Thorac Cardiovasc Surg.

[25] PARK I, CHUNG S (2024). Outcomes of Concomitant Maze Procedure in Tricuspid repair for severe tricuspid Regurgitation[J]. J Korean Med Sci.

[26] RANKIN JS, GRAU-SEPULVEDA MV ADN (2018).

[27] WANG J, LI S (2020). Catheter ablation or surgical therapy in moderate-severe tricuspid regurgitation caused by long-standing persistent atrial fibrillation. Propensity score analysis[J]. J Cardiothorac Surg.

[28] UKITA K, EGAMI Y (2023). Predictors and outcomes of tricuspid regurgitation improvement after radiofrequency catheter ablation for persistent atrial fibrillation[J]. J Cardiovasc Electrophysiol.

[29] ORTIZ-LEON XA, POSADA-MARTINEZ EL, TREJO-PAREDES MC (2022). Tricuspid and mitral remodelling in atrial fibrillation: a three-dimensional echocardiographic study[J]. Eur Heart J Cardiovasc Imaging.

[30] EMBRECHTS PYPEL (2020). Long-term effect of atrial fibrillation on the evolution of mitral and tricuspid valve regurgitation[J]. Acta Cardiol.

