# Supplementary material for: Sinus rhythm restoration reverses tricuspid regurgitation in patients with atrial fibrillation: a systematic review and meta-analysis
Source: J Cardiothorac Surg. 2024 Jul 2;19:411. doi: 10.1186/s13019-024-02891-9 (PMC11218105; doi:10.1186/s13019-024-02891-9)
Supplement: Supplementary file 1 — Supplementary Material 1 [file 13019_2024_2891_MOESM1_ESM.docx]

**Search strategy**

((((((((((((sinus rhythm) OR (restore sinus rhythm)) OR (restoring sinus rhythm)) OR (restore rhythm)) OR (cardioversion)) OR (Ablation, Radiofrequency)) OR (Radio Frequency Ablation)) OR (Ablation, Radio Frequency)) OR (Radio-Frequency Ablation)) OR (Ablation, Radio-Frequency)) OR (radiofrequency ablation)) AND (((((((((((((((((((((((((Atrial Fibrillations) OR (Fibrillation, Atrial)) OR (Fibrillations, Atrial)) OR (Auricular Fibrillation)) OR (Auricular Fibrillations)) OR (Fibrillation, Auricular)) OR (Fibrillations, Auricular)) OR (Persistent Atrial Fibrillation)) OR (Atrial Fibrillation, Persistent)) OR (Atrial Fibrillations, Persistent)) OR (Fibrillation, Persistent Atrial)) OR (Fibrillations, Persistent Atrial)) OR (Persistent Atrial Fibrillations)) OR (Familial Atrial Fibrillation)) OR (Atrial Fibrillation, Familial)) OR (Atrial Fibrillations, Familial)) OR (Familial Atrial Fibrillations)) OR (Fibrillation, Familial Atrial)) OR (Fibrillations, Familial Atrial)) OR (Paroxysmal Atrial Fibrillation)) OR (Atrial Fibrillation, Paroxysmal)) OR (Atrial Fibrillations, Paroxysmal)) OR (Atrial Fibrillations, Paroxysmal)) OR (Fibrillations, Paroxysmal Atrial)) OR (Paroxysmal Atrial Fibrillations))) AND (((functional tricuspid regurgitation) OR (secondary tricuspid regurgitation)) OR ((((((((((((Insufficiency, Tricuspid Valve) OR (Valve Insufficiency, Tricuspid)) OR (Tricuspid Valve Regurgitation)) OR (Regurgitation, Tricuspid Valve)) OR (Valve Regurgitation, Tricuspid)) OR (Tricuspid Valve Incompetence)) OR (Incompetence, Tricuspid Valve)) OR (Valve Incompetence, Tricuspid)) OR (Tricuspid Incompetence)) OR (Incompetence, Tricuspid)) OR (Tricuspid Regurgitation)) OR (Regurgitation, Tricuspid)))

**Supplementary Figure 1.** Funnel plots of (A) Tricuspid regurgitation area, (B) Tricuspid valve annular diameter, (C) Right atrial volume index, (D) Pulmonary artery systolic pressure, (E) Left ventricular ejection fraction, (F) Tricuspid regurgitation severity.


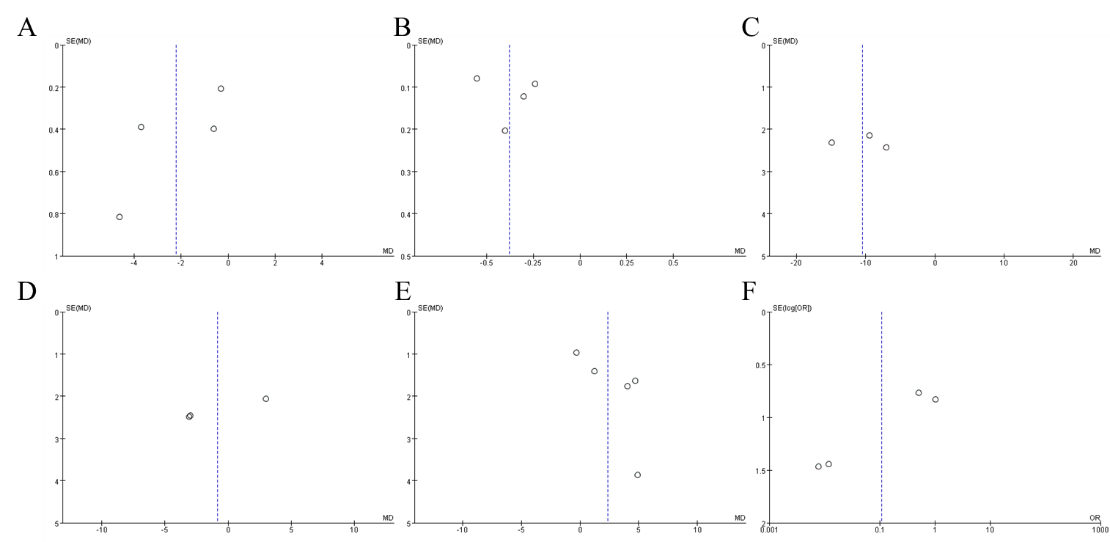


**Supplementary Table 1. Newcastle-Ottawa quality assessment scale.**

| **Author, Ref ^#^** | **Selection** | | **Comparability** | | **Outcome** | |
| --- | --- | --- | --- | --- | --- | --- |
|  | **Points (max 4)** | **Risk of bias** | **Points (max 2)** | **Risk of bias** | **Points (max 3)** | **Risk of bias** |
| Markman TM et al. (13) | 2 | Medium | 1 | Medium | 2 | Medium |
| Nishiwaki S et al. (12) | 3 | Medium | 1 | Medium | 3 | Low |
| Pype L et al. (30) | 2 | Medium | 1 | Medium | 2 | Medium |
| Itakura K et al. (14) | 3 | Medium | 1 | Medium | 2 | Medium |
| Nakatsukasa T et al. (15) | 3 | Medium | 1 | Medium | 2 | Medium |
| Soulat-Dufour L et al. (16) | 4 | Low | 2 | Low | 3 | Low |

Two independent reviewers undertook quality assessment and allocated stars/points for adherence to following criteria:

• Selection (adequate selection and definition of groups)

• Comparability (comparability of two groups for a selected variable and comparability for other variables)

• Outcome (modality of assessment, enough length of follow-up and adequacy of follow-up)

Studies with 4 stars for selection, 2 for comparability, and 3 for outcome were defined at low risk of bias. Studies with 2 or 3 stars for selection, 1 for comparability, and 2 for outcome were defined at medium risk. Any study with a score of 1 for selection or outcome ascertainment, or 0 for any of the three domains, was deemed at high risk of bias.
